# Analysis of Clinical Characteristics and Risk Factors of Severe Adenovirus Pneumonia in Children

**DOI:** 10.3389/fped.2021.566797

**Published:** 2021-10-12

**Authors:** Haiqin Zhong, Xiaoyan Dong

**Affiliations:** Department of Respiratory Medicine, Shanghai Children's Hospital, Shanghai Jiao Tong University, Shanghai, China

**Keywords:** adenovirus, pneumonia, risk factors, children, clinical characteristics

## Abstract

**Objective:** To analyze the clinical characteristics of adenovirus pneumonia (ADVP) in children and explore risk factors for severe ADVP.

**Methods:** Clinical data from 7,008 hospitalized children with community-acquired pneumonia and 211 with ADVP were retrospectively analyzed between July 2014 and June 2019. Eighty-six patients were diagnosed with severe pneumonia, and related risk factors were analyzed.

**Results:** ADVP accounts for 3.01% (211/7008) of CAP in hospitalized children. Among 211 patients, 167 (64.9%) children aged 1–5 years old, and the onset was in winter and spring for 126 (59.7%) children. All patients had cough, and 116 (92.8%) patients with mild cases and 82 (95.4%) patients with severe cases had varying degrees of fever. The duration of fever in the severe ADVP group and mild ADVP group was 7.3 and 5.4 days, respectively. The average hospital stays were 9.8 and 5.8 days, respectively. There was no significant difference in the levels of WBC and ESR between the two groups, but the levels of *N*%, CRP, PCT and LDH in children with severe ADVP were significantly higher than those in the mild ADVP group. The univariate analysis showed that there were significant differences between the severe ADVP group and the mild ADVP group in ≥7 days of fever and high IgE (*P* < 0.05). There was no significant difference in sex, age, onset season, mycoplasma infection, bacterial infection between the two groups (*P* > 0.05). The multivariate logistic analysis showed that ≥7 days of fever and high IgE were independent risk factors for severe ADVP (*P* < 0.05).

**Conclusions:** Children with severe ADVP have long fever duration, a strong inflammatory response and immune function disturbance. Fever duration (≥7 days) and high IgE were independent risk factors for severe ADVP.

## Introduction

Human adenovirus is well known pathogens that cause a variety of human illness, including upper respiratory tract illness, pneumonia, conjunctivitis, gastroenteritis and cystitis (1). Adenovirus (ADV), a nonenveloped double-stranded DNA virus, is known to have more than 90 genotypes, with seven subgroups (A to G) (2). Adenovirus pneumonia (ADVP) is a common respiratory infectious disease in children, accounting for 4–10% of pneumonia cases in children (3). One-third of adenoviral pneumonia develops into severe pneumonia (4). Severe ADVP is characterized by respiratory system involvement, multiple system complications, high mortality, chronic airway and lung diseases, and severe mental and economic burden to family and society. In the present article, we report the characteristics of ADVP and provide an analysis of the clinical features and risk factors associated with severe ADVP cases in children.

## Methods

We conducted a retrospective case-control study combined with a descriptive analysis of clinical data and outcome among children admitted to department of respiratory medicine of Shanghai Children's Hospital over the period from July 2014 and June 2019. Previously healthy 7,008 male and female children between 1 month and 14 years who had been hospitalized for community-acquired pneumonia (CAP) were included in the study. ADVP was defined as clinician-diagnosed pneumonia and positive adenovirus antigen or IgM antibodies. The exclusion criteria of the study were asthma, endotracheal foreign body, known or suspected active tuberculosis, lung mycosis, pulmonary parasitoses, immunodeficiency, receiving immunosuppressive agents. Patient characteristics, including demographics, clinical presentation, laboratory examination and outcomes were extracted from electronic medical records.

Community-acquired pneumonia was defined as fever and cough with chest radiographic findings of lobar/bronchopneumonia or focal infiltrates. Fever was defined as a body temperature of >37.5°C. The duration of fever before hospitalization was provided by the caregivers, whereas the duration of fever after hospitalization was collected from medical records. The diagnosis of severe pneumonia was any one of the following: sustained high fever (>39°C) for more than 5 days, accompanied by a frequent and severe irritating cough; Respiratory rate ≥70 breaths/min (infant), Respiratory rate ≥ 50 breaths/min (≥1 year old); Severe respiratory failure (PaO_2_/FiO_2_ < 250); Rapidly progressing lung shadow with multiple- or single-lobar/segment consolidation; Requirement for mechanical ventilation; Requiring intensive care. Admission to ICU was also based on clinical assessment by pediatric senior medical staff and was indicated if there was clinically significant respiratory distress, dehydration, hypotension and/or suspicion of encephalopathy.

Adenovirus detection was done *via* (1) Adenovirus antigen testing: respiratory tract samples, including tracheal aspirates, bronchoalveolar lavage, induced sputum and nose swabs or nasopharyngeal aspirates, were collected and adenovirus antigen was detected by immunofluorescence test (D3 Ultra DFA Respiratory Virus Screening & ID Kit, Diagnostic Hybrids, Inc.). (2) Specific IgM antibody detection: Two ml of venous blood were taken, and serum specific IgM antibodies of common respiratory pathogens were detected by indirect immunofluorescence assay (Respiratory Tract Profile (IgM), EUROIMMUN Medical Diagnostics (China) Co., Ltd.). In addition, other microbiologic tests for bacteria, viruses and mycoplasma pneumoniae were requested when clinically indicated.

Statistical analysis was performed using the SPSS statistical software program, version 20 (IBM Corporation, Armonk, NY, USA). The measurement data are expressed as the mean ± standard deviation ($\bar{x}$ ± s). The count data were expressed as a percentage or rate. The chi-square test was used for categorical data, and the *t*-test and Mann–Whitney *U* test were used for continuous data. Binary logistic regression analysis was used to examine risk factors that were significant in the univariate analysis. *P* < 0.05 was considered statistically significant.

## Results

### General Information

A total of 7,008 hospitalized children with CAP were investigated and 211 children with ADVP were included in the retrospective study according to the inclusion criteria. ADVP accounts for 3.01% (211/7,008) of CAP in hospitalized children. Among 211 children with ADVP, the ratio was of boys and girls 1.25:1(117:94). The age of onset was 3 months to 8 years. Thirty patients (14.2%) were under 1 year of age, 76 patients (36.0%) were 1–3 years old, 61 patients (26.2%) were 3–5 years old, and 44 patients (20.9%) were older than 5. The cases occurred throughout the year, including 57 cases (27.0%) in spring (March to May), 53 (25.1%) in summer (June to August), 33 (15.7%) in autumn (September to November), and 68 (32.2%) in winter (December to January). Winter and spring were the peak seasons (*n* = 125,59.2%). A total of 86 patients (37.7%) were diagnosed with severe pneumonia, and 125 (62.3%) were diagnosed with mild pneumonia. There was no significant difference in sex, age or onset season between the two groups.

### Clinical Features

Varying degrees of fever were found for 116 (92.8%) patients with mild ADVP and 82 (95.4%) patients with severe ADVP. The duration of fever in the mild groups lasted <10 days for 95 patients (76.0%) and longer than 10 days for 21 patients (16.8%). The duration of fever in the severe groups lasted <10 days for 58 patients (67.4%) and longer than 10 days for 24 patients (27.9%). The average durations of fever for the severe ADVP group and mild ADVP group were 7.3 and 5.4 days, respectively. The average hospital stays were 9.8 and 5.8 days, respectively. The duration of fever and hospital stays of the mild ADVP group were less than those of the severe group.

All patients had cough, and 45 patients (21.3%) also had wheezing. Moreover, 56 patients (65.1%) had wet rales, and 21 patients with severe ADVP (24.4%) had a wheezing sound in their lungs. 75 patients (60.0%) had wet rales, and 24 patients with mild ADVP (19.2%) had a wheezing sound.

### Laboratory Tests

There was no significant difference in the white blood cell count (WBC) and erythrocyte sedimentation rate (ESR) between the two groups, but the neutrophil (N%), C-reactive protein (CRP), procalcitonin (PCT) and lactate dehydrogenase (LDH) levels in children with severe ADVP were significantly higher than those in the mild ADVP group, as shown in Table 1.

**Table 1:** Comparison of inflammatory indicators between the mild ADVP group and the severe ADVP group.

**Items**	**Mild ADVP**	**Severe ADVP**	***t/Z value***	***P***
WBC (×10^9^/L)^a^	9.15 ± 5.10	9.68 ± 5.13	0.734	0.464
N%^a^	48.79 ± 19.10	55.74 ± 20.33	2.529	0.012^*^
CRP (mg/L)^a^	19.39 ± 19.32	28.49 ± 38.05	2.283	0.023^*^
ESR (mm/h)^a^	44.91 ± 26.83	47.72 ± 28.28	0.731	0.466
PCT (ng/mL)^b^	0.52 ± 1.08	1.69 ± 4.88	−2.484	0.013^*^
LDH (μmol/L)^b^	333.12 ± 94.09	563.11 ± 424.29	−6.005	0.000^*^

a *t-text;*

b *Mann-Whitney U test;*

* *P < 0.05*.

### Radiographic Examination

Chest X-ray examination was performed in all 211 children, and patchy opacity (177 cases, 83.9%), patchy consolidation (118 cases, 55.9%) and pleural effusion (70 cases, 33.2%) were most common.

Chest CT examination was performed in 86 severe ADVP cases, and patchy opacity of bilateral lungs (80 cases, 93.0%), lung consolidation and/or atelectasis (67 cases, 77.9%) were most common. The range of pulmonary lesions involved one lobe in eight cases (9.3%), six lobes in two cases (7.0%), more than or equal to three lobes in 72 cases (83.7%).

### Risk Factors for Severe ADVP

#### The Univariate Analysis

There were significant differences between the severe ADVP group and the mild ADVP group in ≥7 days of fever and high IgE (*P* < 0.05). There was no significant difference in sex, age, onset season, mycoplasma infection, bacterial infection, between the two groups (*P* > 0.05), as shown in Table 2.

**Table 2:** Univariate analysis between the mild ADVP group and the severe ADVP group.

**Characteristics**		**Mild ADVP (*n* = 125)**	**Severe ADVP (*n* = 86)**	**χ^2^** ***value***	***P*** ***^a^***
Sex	Male	67	50	0.425	0.514
	Female	58	36		
Age	≤ 1 years	16	14	0.808	0.847
	1~3 years	44	32		
	3~5 years	38	23		
	>5 years	27	17		
Onset season	Spring	33	25	2.397	0.494
	Summer	32	20		
	Autumn	23	10		
	Winter	37	31		
Days of fever	≥7	39	43	7.579	0.006^*^
	<7	86	43		
Mycoplasma infection	Yes	57	40	0.017	0.896
	No	68	46		
Bacterial infection	Yes	23	19	0.436	0.509
	No	102	67		
High IgE	Yes	63	58	6.049	0.014^*^
	No	62	28		

a *chi-square tests;*

* *P < 0.05*.

#### Multivariate Logistic Analysis

Binary logistic regression analysis was used to examine risk factors that were significant in the univariate analysis. The independent variables were assigned values: ≥7 days of fever: No = 0, Yes = 1; high IgE: No = 0, Yes = 1. Whether severe ADVP occurred or not was taken as the response variable. The results showed that ≥7 days of fever and high IgE were independent risk factors for severe ADVP (*P* < 0.05), as shown in Table 3.

**Table 3:** Multivariate logistic analysis of severe ADVP.

**Independent variables**	***B***	***SE***	***Wald*** **χ^2^**	***df***	***P***	***OR***	**95%** ***CI***
≥7 days of fever	0.801	0.294	7.412	1	0.006^*^	2.227	1.251~3.964
High IgE	0.723	0.297	5.927	1	0.015^*^	2.061	1.151~3.689

* *P < 0.05*.

## Discussion

ADV is one of the severe types of community-acquired pneumonia in children. ADVP accounts for 3.01% of CAP in hospitalized children in our study. The early clinical manifestations of ADVP lack specificity, and most of them begin with fever and cough. It is difficult to distinguish from other respiratory tract infectious diseases, and relevant pathogenic detection methods are needed. A total of 92.8% of mild and 95.4% of severe cases of ADVP were associated with varying degrees of fever. Patients with severe cases had a longer duration of fever. The disease progresses rapidly in a short period of time. Symptoms and signs of the respiratory system, including cough, wheezing, and rales, appeared obviously. Early etiological detection is the main method for the diagnosis of ADVP.

ADVP is more likely to occur in children aged 6 months to 5 years. As infants and young children grow, the number of maternal antibodies gradually decreases in the body, but their respiratory and immune systems are still not fully developed. Moreover, low local mucosal immunity contributes to a high incidence of adenoviral pneumonia in this age group (5). In this study, 79.1% of the children were under 5 years of age, which was consistent with domestic and foreign reports (6, 7). However, there was no significant difference in age between the two groups. Moreover, the two groups showed no significant difference in the onset season of ADVP, which was relatively high in winter and spring. Duan et al. (8) investigated the prevalence of ADV infection in children with community-acquired pneumonia (CAP) in 12 hospitals in nine provinces (municipalities directly under the central government) in China, and the incidence of ADV infection peaked in children aged l~3 years in Northern China, while there was no significant difference in the positive detection rate among age groups in Southern China. The season with the highest ADV detection rate in northern China was winter, and in southern China, it was spring and summer, suggesting that the onset ages and peak seasons may vary from region to region.

The levels of N%, CRP, PCT and LDH in children with severe ADVP were significantly higher than those in the mild ADVP group. The relationship between N%, CRP, PCT and the severity of ADVP has been confirmed by many studies (9–12). LDH is found in all animal tissues, and the highest activity is found in the liver, followed by the heart, skeletal muscle, kidney, lung and tumor. When the lung tissue is obviously damaged, the serum LDH level could be increased. In many diseases, it is a reliable indicator of severity and prognosis. Wen et al. (13) showed that serum LDH was higher in children with respiratory infection than in the healthy control group and was also higher in the pneumonia group than in the upper respiratory infection group. This study suggested that LDH elevation may be useful for the early identification of severe ADVP. In addition, Patel et al. (14) observed that adenovirus causes a greater increase in TNF-α than other viruses. Sun et al. (15) found a significant increase in IL-6 levels in children with severe adenovirus infection, which was positively correlated with the severity of infection. Zheng et al. (16) found that the higher the level of IL-8 in children with ADVP, the worse the prognosis was. Therefore, there is a strong inflammatory process in severe ADVP.

Mixed mycoplasma infection in patients with ADVP was more common than mixed bacterial infection. But there was no significant difference in mycoplasma infection and bacterial infection between the two groups. The results indicated that for children with ADVP in this area, the occurrence of severe pneumonia is related to their physical fitness and immune function. Considering that children with poor physical fitness and low immune function are more likely to have mixed infection, the mixed infection was balanced in the two groups.

Literature has reported that the imaging manifestations of adenovirus pneumonia in children are mainly multiple segmental consolidation, atelectasis and patchy opacity in both lungs (17). More than three lobes of pulmonary lesions are involved in 72 cases (83.7%) of severe ADVP. The main clinical features of cluster consolidation are centripetal distribution and high density. Some clinical studies have shown that the main reason for this clinical feature is related to the basic pathology of ADVP. When a patient is infected with ADV, the ADV will spread to the lung parenchyma along the airway and through the bronchus. When the degree of the lesion involves the bronchioles, it can affect the alveoli of the patient and further develop into lung consolidation.

Both of the univariate and multivariate logistic analysis suggested that there was significant difference in ≥7 days of fever and high IgE between the two groups. Different studies show different risk factors for severe ADVP. It may be related to the different people, regions, environment, climate, and contents of comparisons. Jin et al. (18) found that age from 6 months to 2 years, nutritional anemia and immunodeficiency were independent risk factors for severe ADVP. Huang et al. (19) found that congenital airway dysplasia, history of previous surgery, and hemoglobin <90 g/L in children with ADVP had a greater association with severe disease. Xie et al. (20) showed that HADV load, age, and fever duration were risk factors for ADVP severity. Cheng et al. (21) demonstrated that patients who present with underlying neurologic diseases, chronic lung diseases, and malnutrition have higher incidences of ADVP and that ADV-7 infection more frequently develops into severe pneumonia. Overall, children with young age, long fever duration, underlying diseases and complications are more likely to develop severe ADVP.

At this point, the mechanism of severe adenovirus infection is unknown, although it is believed to be related to ADV itself and the ADV-induced inflammatory response and immune dysfunction. In this study, the IgE levels of ADVP were different in the mild and severe ADVP groups, and the severity of pneumonia was positively correlated with the IgE levels of ADVP. Abnormally high IgE levels are thought to be a marker of immune disorders (22, 23). At present, there are few studies on the correlation between ADV infection and IgE levels. Adenovirus, Mycoplasma pneumoniae, influenza virus and other infections could increase serum total IgE levels in children with respiratory tract infection (24). IL-4 promotes the production of IgG and IgE by B cells, so an abnormally high level of IL-4 may increase the IgE level in children with adenovirus infection (25), which may lead to inflammatory changes in the respiratory system. Yang et al. (26) showed that IgE and dsDNA-IgE in BALF may contribute to lung injury caused by HADVs, especially in severe cases and elevated dsDNA-IgE may serve as an indicator of severity in children with HADVs pneumonia. Ye et al. (27) demonstrated that the P1 protein of Mycoplasma pneumoniae can induce the production of P1-specific IgE. Zhang et al. (28) found that the severity of respiratory syncytial virus (RSV) bronchitis in children was positively correlated with serum IgE level. The complexity of immunopathology and individual differences mean that ADV infection in children present a variety of disease processes, and immune dysfunction is an important pathogenesis that affects prognosis. We should pay more attention to the disease condition of ADVP patients with high IgE and select glucocorticoid or gamma globulin for immunoregulatory intervention therapy while controlling infection.

Our study also had limitations. First, the type of adenovirus is related to the severity of the disease and it was not detected in this retrospective study. Early identification is helpful for clinicians to identify severe cases and avoid delayed treatment. Second, this study discussed the correlation with IgE in acute infections and imply IgE was elevated in ADV infection. The results needed to be validated in more clinical cases and animal experiments. Besides, IL-4 may increase the IgE level. Data on IL-4 in patients were lacking in this study. Therefore, in the subsequent study, we could collect more data and analyze the relationship between the IgE level and IL-4, and it is best to study the mechanism involved on molecular biology level.

## Conclusions

Severe ADVP has a strong inflammatory reaction process, and immune dysfunction. Fever duration (≥7 days) and high IgE are risk factors for severe ADVP. The detection of serum total IgE in children with ADVP not only provides a basis for the diagnosis and treatment of secondary allergic disease in children but also helps to judge the severity of the disease in order to provide early immune intervention and reduce inflammatory reactions. More attention should thus be paid to such cases.

## Data Availability Statement

The original contributions presented in the study are included in the article/supplementary material, further inquiries can be directed to the corresponding author.

## Ethics Statement

The studies involving human participants were reviewed and approved by the Ethics Committee of Shanghai Children's Hospital. Written informed consent from the participants' legal guardian/next of kin was not required to participate in this study in accordance with the national legislation and the institutional requirements.

## Author Contributions

HZ collected the patient's information, summarized the data, and wrote the paper. XD was a major contributor in revising the manuscript. Both authors read and approved the final manuscript.

## Conflict of Interest

The authors declare that the research was conducted in the absence of any commercial or financial relationships that could be construed as a potential conflict of interest.

## Publisher's Note

All claims expressed in this article are solely those of the authors and do not necessarily represent those of their affiliated organizations, or those of the publisher, the editors and the reviewers. Any product that may be evaluated in this article, or claim that may be made by its manufacturer, is not guaranteed or endorsed by the publisher.

## References

[1] Radke JR Cook JL. Human adenovirus infections: update and consideration of mechanisms of viral persistence. Curr Opin Infect Dis. (2018) 31:251-6. 10.1097/QCO.0000000000000451 29601326 PMC6367924

[2] Jobran S Kattan R Shamaa J Marzouqa H Hindiyeh M. Adenovirus respiratory tract infections in infants: a retrospective chart-review study. Lancet. (2018) 391:S43. 10.1016/S0140-6736(18)30409-4 29553443

[3] Fu FX Tang Z Ye Z Mo S Tian X Ni K. Human adenovirus type 7 infection causes a more severe disease than type 3. BMC Infect Dis. (2019) 19:36. 10.1186/s12879-018-3651-2 30626350 PMC6327436

[4] Lu MP Ma LY Zheng Q Dong LL Chen ZM. Clinical characteristics of adenovirus associated lower respiratory tract infection in children. World J Pediatr. (2013) 9:346–9. 10.1007/s12519-013-0431-3 24235068

[5] Berciaud S Rayne F Kassab S Jubert C Corte MF Salin F. Adenovirus infections in Bordeaus University Hospital 2008-2010: clinical and virological features. J Clin Virol. (2012) 54:302–7. 10.1016/j.jcv.2012.04.009 22608365

[6] Liu Q Xie LY Zhang B Zhong LL Yu T Zeng SZ. Epidemiological investigation of adenovirus pneumonia in children in Hunan province. Chin Pediatr Emerg Med. (2019) 26:752–7. 10.3760/cma.j.issn.1673

[7] Li L Woo YY de Bruyne JA Nathan AM Kee SY Chan YF. Epidemiology, clinical presentation and respiratory sequelae of adenovirus pneumonia in children in Kuala Lumpur, Malaysia. PLoS ONE. (2018) 13:e0205795. 10.1371/journal.pone.0205795 30321228 PMC6188781

[8] Duan YL Zhu Y Xu BP Li CC Chen AH Deng L. Multicenter study of human adenovirus infection in pediatric community-acquired pneumonia in China. Chin J Pediatr. (2019) 57:27–32. 10.3760/cma.j.issn.0578-1310.2019.01.008 30630228

[9] Rajkumar V Chiang CSM Low JM Cui L Lin RTP Tee NWS. Risk factors for severe adenovirus infection in children during an outbreak in Singapore. Ann Acad Med Singap. (2015) 44:50–9. 25797817

[10] Chen J Chen X Tan XR Liu DH Li XF Zhang XX. Adenovirus pneumonia with hemophagocytic lymphohistiocytosis in children: a clinical analysis of 7 children. Zhongguo Dang Dai Er Ke Za Zhi. (2020) 22:749–54. 10.7499/j.issn.1008-8830.1912148 32669173 PMC7389613

[11] Liu JL Xu F Zhou H Wu XJ Shi LX Lu RQ. Expanded CURB-65: a new score system predicts severity of community-acquired pneumonia with superior efficiency. Sci Rep. (2016) 6:22911. 10.1038/srep22911 26987602 PMC4796818

[12] Zhydkov A Christ-Crain M Thomann R Hoess C Henzen C Werner Z. Utility of procalcitonin, C-reactive protein and white blood cells alone and in combination for the prediction of clinical outcomes in community-acquired pneumonia. Clin Chem Lab Med. (2015) 53:559–66. 10.1515/cclm-2014-0456 25014522

[13] Wen CB Luo B Liu QZ Liang JL. Changes of serum uric acid, lactate dehydrogenase and alpha-hydroxybutyrate dehydrogenase in children with respiratory tract infection. China Medicine and Pharmacy. (2019) 9:65–7. 10.3969/j.issn.2095-0616.2019.04.022

[14] Patel JA Nair S Ochoa EE Huda R Roberts NJ Chonmaitree T. Interleukin-6^−174^ and tumor necrosis factor α^−308^ polymorphisms enhancecytokine production by human macrophages exposed to respiratory viruses. J Interferon Cytokine Res. (2010) 30:917–21. 10.1089/jir.2010.0033 20973681 PMC3004134

[15] Sun J Xiao Y Zhang M Ao T Lang S Wang J. Serum inflammatory markers in patients with adenovirus respiratory infection. Med Sci Monit. (2018) 24:3848–55. 10.12659/MSM.910692 29877315 PMC6020746

[16] Zheng LL Xu HY Ying DH. T. lymphocyte subset level in children with severe adenoviral pneumonia and its clinical significance. Zhejiang Medical Journal. (2018) 40:1702–4. 10.12056/j.issn.1006-2785.2018.40.15.2018-591

[17] Han BK Son JA Yoon HK Lee SL. Epidemic adenoviral lower respiratory tract infection in pediatric patients: radiographic and clinical characteristics. AJR Am J Roentgenol. (1998) 170:1077–80. 10.2214/ajr.170.4.9530062 9530062

[18] Jin DD Zhou WF Li Y Kong XX Tian JM. Characteristics of mixed infection of adenovirus pneumonia in children and risk factors of severe cases. J Clini Pulmon Med. (2019) 24:1747–50. 10.3969/j.issn.1009-6663.2019.10.001

[19] Huang CG Chen L Song YD. Characteristics and risk factors of infection with other pathogens of severe adenovirus pneumonia in children. J Hebei Medical University. (2019) 40:1221–5. 10.3969/j.issn.1007-3205.2019.10.025

[20] Xie L Zhang B Zhou J Huang H Zeng S Liu Q. Human adenovirus load in respiratory tract secretions are predictors for disease severity in children with human adenovirus pneumonia. Virol J. (2018) 15:123. 10.1186/s12985-018-1037-0 30086789 PMC6081882

[21] Cheng JL Peng CC Chiu NC Weng LC Chiu YY Chang L. Risk Factor Analysis and molecular epidemiology of respiratory adenovirus infections among children in Northern Taiwan, 2009-2013. J Microbiol Immunol Infect. (2017) 50:418–26. 10.1016/j.jmii.2015.08.006 26454422

[22] Williams KW Milner JD Freeman AF. Eosinophilia associated with disorders of immune deficiency or immune dysregulation. Immunol Allergy Clin North Am. (2015) 35:523–44. 10.1016/j.iac.2015.05.004 26209898 PMC4688016

[23] Magen E Schlesinger M David M Ben-Zion I Vardy D. Selective IgE deficiency, immune dysregulation, and autoimmunity. Allergy Asthma Proc. (2014) 35:e27–33. 10.2500/aap.2014.35.3734 24717782

[24] Liu HJ Dou M Chen H Chen J Pan XJ Shen LS. The correlation between atypical respiratory pathogens infection and serum total IgE levels. Int J Lab Med. (2015) 36:2480–4. 10.3969/j.issn.1673-4130.2015.17.011

[25] Li Y Gao Q Yuan X Zhou M Peng X Liu X. Adenovirus expressing IFN-λ1 (IL-29) attenuates Allergic airway inflammation and airway hyperreactivity in experimental asthma. Int Immunopharmacol. (2014) 21:156–62. 10.1016/j.intimp.2014.04.022 24819718

[26] Yang DY Lu BT Shi TT Fan HF Zhang DW Huang L. Total and double-stranded DNA-specific immunoglobulin E in bronchoalveolar lavage fluid of children with human adenovirus pneumonia. J Infect Chemother. (2020) 26:986–91. 10.1016/j.jiac.2020.04.024 32473848

[27] Ye Q Mao JH Shu Q Shang SQ. Mycoplasma pneumoniae induces allergy by producing P1-specific immunoglobulin E. Ann Allergy Asthma Immunol. (2018) 121:90–9. 10.1016/j.anai.2018.03.014 29555351

[28] Zhang RF Liang X Chen D Guo L Yang H Xu Y. Expression levels and clinical significance of serum 25 (OH) D3 and immunoglobulin E in children with respiratory syncytial virus bronchiolitis. International Journal of Laboratory Medicine. (2019) 40:162–5. 10.3969/j.issn.1673-4130.2019.02.009

